# Corrosion Behavior of Bubble Tubes in Glass Curing Furnaces Under the Heat–Flow Coupling Effect

**DOI:** 10.3390/ma19112429

**Published:** 2026-06-05

**Authors:** Heyi Guo, Ce Zheng, Yingjv Li, Qiuyan Huang, Qingbin Zhao, Minhang Sun, Yuansheng Yang

**Affiliations:** 1 Verification Technology Center, China Nuclear Power Engineering Co., Ltd., Beijing 100840, China; guohyagl@163.com (H.G.); qbinzhao@163.com (Q.Z.); minhangs@126.com (M.S.); 2 Institute of Metal Research, Chinese Academy of Sciences, Shenyang 110016, China; yjli@imr.ac.cn (Y.L.); ysyang@imr.ac.cn (Y.Y.)

**Keywords:** vitrification process, Joule-heated ceramic melter, bubbling tube, thermo-flow coupling, high-temperature corrosion

## Abstract

The bubble tube of a glass curing furnace was subjected to extreme heat–flow coupling conditions for a long time due to the scouring of melt flow caused by the gas flow bubbling in a high-temperature molten glass environment at 1150 °C, resulting in severe corrosion and structural failure. This paper conducts post-service sampling analysis of an Inconel 690 bubble tube, and systematically studies its corrosion morphologies, product distribution and corrosion mechanisms. The results show that the outer wall of the bubble tube undergoes an oxidation reaction in the high-temperature molten glass to form a Cr-rich oxide layer. However, local spalling occurs under the scouring of the molten glass flow, resulting in continuous corrosion. The corrosion behavior shows obvious asymmetry. The average corrosion rate near the bubble flow side (the inner curve side, 0.118 mm/day) is significantly higher than that on the outer side (0.051 mm/day) due to the higher partial pressure of oxygen and greater flow rate of molten glass. It reveals the synergistic mechanism by which fluid scouring continuously removes the protective Cr-rich oxide scale, thereby accelerating the oxidation–erosion cycle under the heat-flow coupling effect. The results provided experimental evidence and theoretical reference for the material optimization and life prediction of bubble tubes.

## 1. Introduction

**Figure 1:**
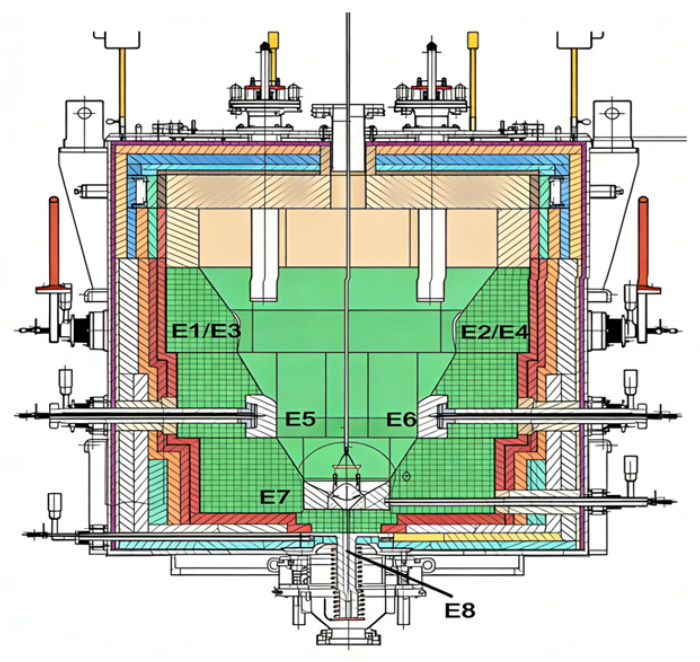
Schematic diagram of Joule-heated ceramic melter.

The nuclear industry and the safe disposal of the highly radioactive and toxic wastes it generates are key links in ensuring the sustainable development of the industry and the safety of the ecological environment [1,2,3]. Vitrification technology, particularly using borosilicate glass systems (typically containing approximately 55–60 wt.% SiO_2_ and 15–20 wt.% B_2_O_3_, along with Na_2_O, Al_2_O_3_, and other network modifiers), offers advantages such as high chemical stability of the solidified bodies, low leaching rates (typically < 10^−3^ g·m^−2^·d^−1^ for simulated waste forms), and good long-term safety. These characteristics have established it as the internationally recognized preferred technology for the treatment of high-level radioactive waste [4,5,6,7]. The core component of this vitrification system is the Joule-heated ceramic melter (Figure 1), whose operational stability directly determines the efficiency and long-term safety of waste treatment [8,9,10]. The Joule-heated ceramic melter (Figure 1) consists of a refractory-lined vessel containing the borosilicate glass melt at 1150 °C. The furnace is equipped with heating electrodes (E1–E8) to maintain temperature stability and a pouring spout for periodic discharge of the vitrified product. The bubbling tube, as a key functional component of the furnace bubbling system, achieves forced stirring of the melt by introducing inert gases (such as argon) or air into the high-temperature molten glass liquid to form a gas–liquid two-phase flow. This process intensifies heat and mass transfer, and promotes the melting of raw materials and homogenization of components. At the same time, it helps to remove bubbles from the molten glass, thereby enhancing the uniformity and chemical stability of the final glass.

However, the service environment of the bubbling tube is extremely harsh. On one hand, it is immersed for long periods in molten glass at temperatures of 1150–1200 °C or higher, at which the glass melt is highly chemically reactive [11,12,13]. On the other hand, the intense flow of the melt caused by gas bubbling creates a continuous fluid shear and dynamic scouring of the tube wall. This combined effect of “high-temperature corrosion” and “fluid scouring” leads to widespread severe corrosion failure in bubbling tubes, especially in the head area immersed in the melt, mainly manifested as non-uniform thinning of the tube wall, local perforation, surface spalling and significant reduction in head diameter [14]. Failure not only leads to unplanned downtime of equipment and a sharp increase in maintenance costs, but also may cause radioactive or toxic leakage, posing a serious safety risk.

At present, research on the corrosion behavior of materials in glass curing furnaces is mainly focused on static or quasi-static contact components such as furnace lining refractory materials or heating electrodes [15,16,17,18,19,20,21]. For example, Gan et al. [20] earlier studied the corrosion of the Inconel 690 alloy in glass melts and found that a composite oxide film was formed on its surface with an outer layer of Fe-Ni spinel phase and an inner layer of Cr_2_O_3_, with a thickness ranging from 5 to 100 μm, but the dissolution of the oxide film in the melt limited its long-term protection. Halder et al. [21] studied the static corrosion of Inconel 693 in borosilicate glass and found that the formation of a continuous internal Al_2_O_3_ layer could effectively suppress the generation and outward diffusion of harmful spinel phases.

In addition, recent studies have increasingly recognized the importance of coupled degradation mechanisms in various engineering systems. For instance, Zeng et al. [22] investigated flow-accelerated corrosion of X65 steel in gradual contraction pipes under high CO_2_ partial pressure, demonstrating that fluid hydrodynamics significantly enhance corrosion rates by accelerating the rupture of corrosion product scales. Pei et al. [23] studied oxidation-creep damage in Ni-based single-crystal superalloys, revealing the synergistic interaction between high-temperature oxidation and mechanical loading. Meng et al. [24] examined microstructural modification and stress corrosion mechanisms in friction stir welding joints, showing how severe plastic deformation influences corrosion susceptibility. Similarly, research on selective laser melting manufactured 316 L stainless steel in simulated body fluids [25] has demonstrated that manufacturing-induced microstructural features can substantially alter corrosion behavior. These studies collectively highlight that coupled environments—involving simultaneous chemical, mechanical, and flow effects—produce degradation phenomena that cannot be predicted from isolated testing. However, for the specific case of bubble tubes in glass curing furnaces subjected to intense “thermal–flow” coupling, systematic investigations remain limited.

Therefore, an in-service bubble tube is taken as the research object. Systematic microstructural and compositional analysis is conducted. The corrosion behavior and underlying mechanisms under the heat–flow coupling effect in a molten glass environment are aimed to be clarified. A comprehensive discussion was presented from the perspectives of environmental characteristics, corrosion mechanisms, influencing factors, and protection strategies, with the goal of providing a basis for material selection, lifetime prediction, and maintenance strategies for bubble tubes.

## 2. Materials and Methods

### 2.1. Bubble Tube Samples and Service Conditions

A bubble tube for a Joule-heated ceramic melter was investigated, with a total length of 1581 mm, consisting of a right-angle section at the rear (1380 mm) and an inclined section at the front. The outer diameter of the bubble tube is 30 mm. The inner diameter of the bubble tube is 6 mm, resulting in an initial wall thickness of 12 mm. The material of bubble tube was IN 690 alloy, with a chemical composition of 28–30% Cr, 9–11% Fe, 0.3% Si, 0.2% Mn, 0.02% C and balance Ni (in wt.%). During service, the bubble tube was immersed in a borosilicate glass melt at 1150 °C, and argon gas was continuously introduced into the tube with flow rate of 0.6–1.0 L/min for 30 days’ service. In the end, the bubble tube was removed and the corrosion behaviors were discussed in detail. The service conditions and operating parameters for the bubble tube are summarized in Table 1.

### 2.2. Sampling Preparation

**Figure 2:**
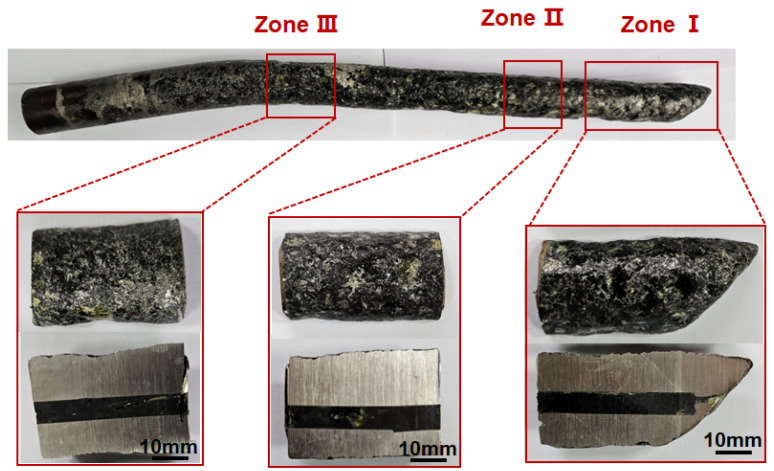
Schematic diagram of bubbling tube sampling locations (Zone I, II, III).

For a comprehensive assessment of the corrosion conditions, sample sections of 30 mm in length were cut along the axial direction of the bubble tube at three typical positions: the head (Zone I, near the outlet), the middle (Zone II), and the tail (Zone III, near the corner) (Figure 2). Each section was cut longitudinally, then ground and polished by sandpaper step by step to 2000#, and polished to a mirror finish with 2.5 μm diamond polishing paste to make microstructure observation specimens.

### 2.3. Microstructure Characterization

The macroscopic morphologies of corrosion and the changes in cross-sectional thickness were preliminarily observed by using a metallographic microscope (OM, Zeiss Axio Observer Z1, Zeiss, Oberkochen, Baden-Württemberg, Germany). High-resolution morphology observations and micro-area composition analysis of the corrosion areas were carried out using a scanning electron microscope (SEM, Inspect F50, FEI, Hillsboro, OR, USA) at an accelerating voltage of 20 kV and a working distance of 10 mm. Meanwhile, an energy-dispersive spectrometer (EDS, Oxford X-maxN, Oxford Instruments, Oxford, UK) was applied with a focus on characterizing the type, distribution, oxide film morphology, thickness and elemental diffusion behavior of the corrosion products. The data acquisition for EDS results was performed at a count rate of approximately 5000 cps with a process time of 5 μs.

The corrosion rates for the bubble tube at three regions were calculated by using Equation (1).

(1) $$v=\frac{d_{0}-d_{1}}{t}$$

*d*_0_ is the initial thickness of the bubble tube (12 mm), *d*_1_ is the thickness of bubble tube after corrosion, and *t* is the corrosion time (30 days).

## 3. Results

### 3.1. Corrosion Rates for Bubbling Tube

The average corrosion rates in the different positions of the bubble tube are shown in Table 2. Obviously, the average corrosion rates for outer side of bubble tube were much smaller than the inner side. For the three zones, the corrosion rate for Zone III was the lowest, followed by Zone I and Zone II.

### 3.2. Corrosion Characteristics for Zone I

Zone I is located at the front end of the bubble tube, which lies at the outlet end, directly immersed in the molten glass. Subjected to the intense thermodynamic coupling of bubble formation and melt flow over an extended period, this region endures the most severe corrosion conditions. Consequently, it becomes the area with the most severe corrosion, based on the corrosion rates results in Table 2. It also exhibits the most representative corrosion morphologies within the entire bubble tube.

**Figure 3:**
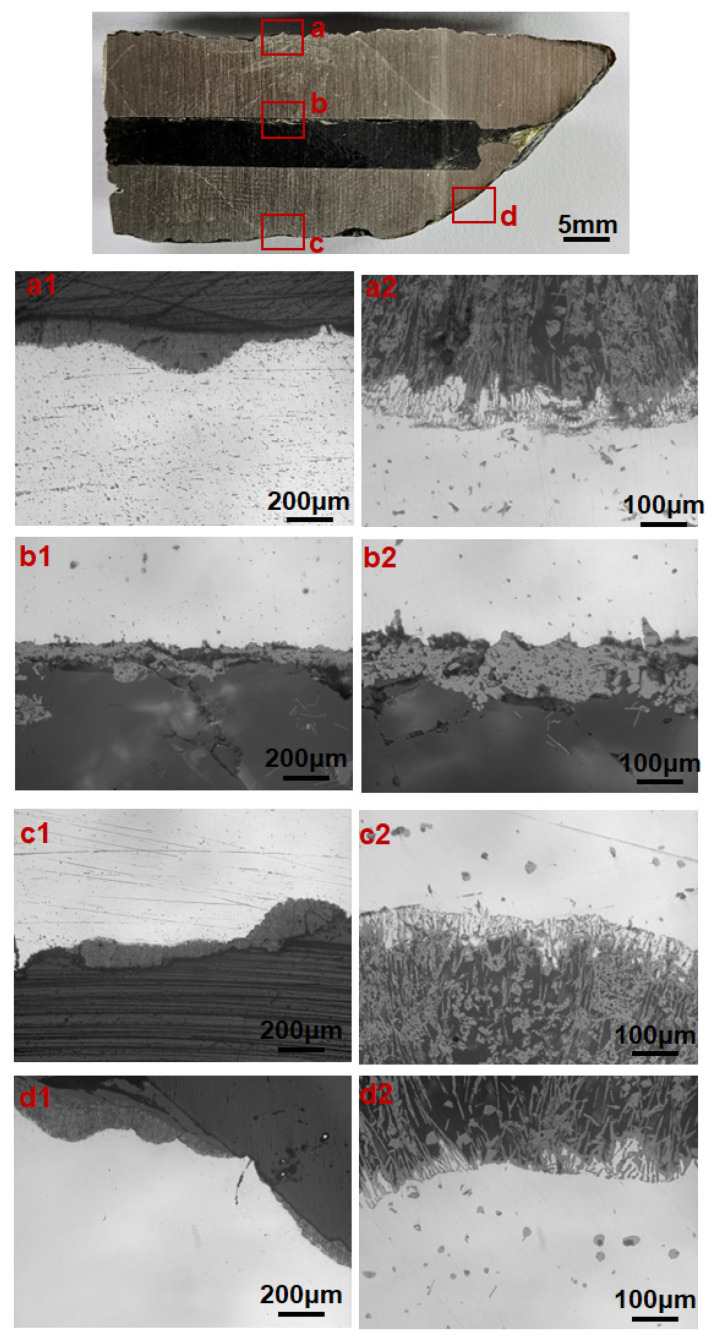
Microstructures for Zone I in bubble tube: (**a**) the outside; (**b**) the inner wall; (**c**) the inner side; (**d**) the outlet for bubble tube. (**a1**,**a2**) microstructures of position (**a**) at different magnifications. (**b1**,**b2**) microstructures of position (**b**) at different magnifications. (**c1**,**c2**) microstructures of position (**c**) at different magnifications. (**d1**,**d2**) microstructures of position (**d**) at different magnifications.

Figure 3 shows the macroscopic morphologies of the bubble tube, which show significant axial asymmetry: the wall thickness is relatively thick in the upper part (outer bend side) and significantly thinner in the lower part (inner bend side). The inner cavity of the tube is filled with borosilicate glass (Figure 3(b1,b2)), indicating that the melt had infiltrated the tube during service. This up-and-down asymmetric corrosion thinning intuitively reflects the gradient in the flow field, temperature field, or oxygen partial pressure distribution in the area, resulting in different rates of material loss.

Further microscopic corrosion morphologies and product distributions are shown in Figure 3(a2–d2). The outer wall surface of the outer curved side (upper half) of the bubble tube is covered with a layer of glass with a tortuous interface (Figure 3(a2,c2)). There are a large number of long, strip-shaped and block-shaped corrosion products at the interface between the matrix (bright white) and the glass (black), which extend into the interior of the glass. A layer of long strip-shaped corrosion products is visible near the interface of the matrix.

**Figure 4:**
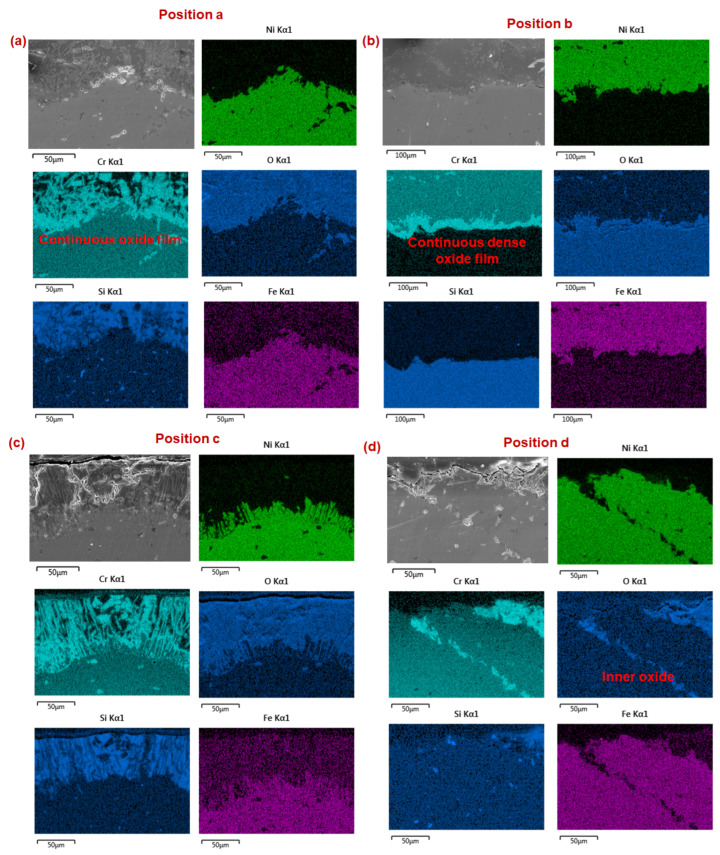
EDS results of the corrosion layers on the bubble tube in Zone I. (**a**) Position a; (**b**) position b; (**c**) position c; (**d**) position d.

Further SEM-EDS analysis (Figure 4) indicated that the needle-like and plate-like products were enriched with Cr and O elements and were identified as Cr-containing oxide. In addition, a relatively continuous Cr-containing oxide film formed at the matrix and glass interface. However, there are still Cr and O enrichment zones in the matrix beneath the oxide film, indicating internal oxidation: oxygen diffuses into the matrix through defects in the oxide film, such as microcracks and pores, and reacts with the solid solution of Cr to form secondary Cr_2_O_3_ [19,26,27]. This indicates that the surface oxide film is not dense enough to completely block the inward diffusion of oxygen.

Position a also shows a thinner continuous Cr–O layer (Figure 4a). The film appears slightly thinner, and the interface with the Ni matrix remains distinguishable. This suggests that while a protective layer has formed, it may possess a less compact microstructure, possibly due to minor variations in local temperature, oxygen activity, or flow conditions. Nevertheless, the continuity of the film still offers considerable protection.

Position b in Zone I exhibits the most protective corrosion morphology (Figure 4b). Here, Cr and O form a uniform and continuous layer across the entire mapped area, with a sharp interface between the oxide film and the underlying matrix. The annotation “continuous dense oxide film” indicates that this layer is both structurally intact and compact. This configuration effectively blocks inward oxygen diffusion, thereby preserving the metallic matrix and representing the ideal outcome of high-temperature oxidation under stable conditions.

At position c (Figure 4c), the elemental maps reveal that Cr and O are enriched only in isolated, discrete regions, while the majority of the surface lacks any continuous coverage. Ni remains relatively uniformly distributed, suggesting that the matrix has not been extensively consumed. This pattern is characteristic of localized or selective oxidation, where an incomplete oxide layer fails to provide uniform protection. Such behavior is often associated with early-stage corrosion or regions where fluid scouring intermittently removes developing oxide scales.

Position d presents a markedly different corrosion pattern (Figure 4d). No continuous Cr–O layer is observed on the surface. Instead, Cr and O enrichments are distributed along paths within the interior of the matrix, characteristic of internal oxidation. Ni depletion is evident in these regions, indicating that the original alloy composition has been compromised. This morphology typically arises when the surface oxide film becomes unstable or is mechanically disrupted, allowing oxygen to diffuse inward along rapid transport pathways such as grain boundaries, where it reacts to form matrix oxides.

### 3.3. Corrosion Characteristics for Zone II

Zone II is located in the transition zone behind the head of the bubbling tube (Figure 2), adjacent to Zone I at the forefront of corrosion. Although still within the range of the strong flow of the melt, the intensity of bubble formation and fluid scouring in this area is slightly milder than that in Zone I. The corrosion morphology shows similar but distinct features of Zone I, reflecting the gradient change in “thermo-fluid–chemical” coupling.

**Figure 5:**
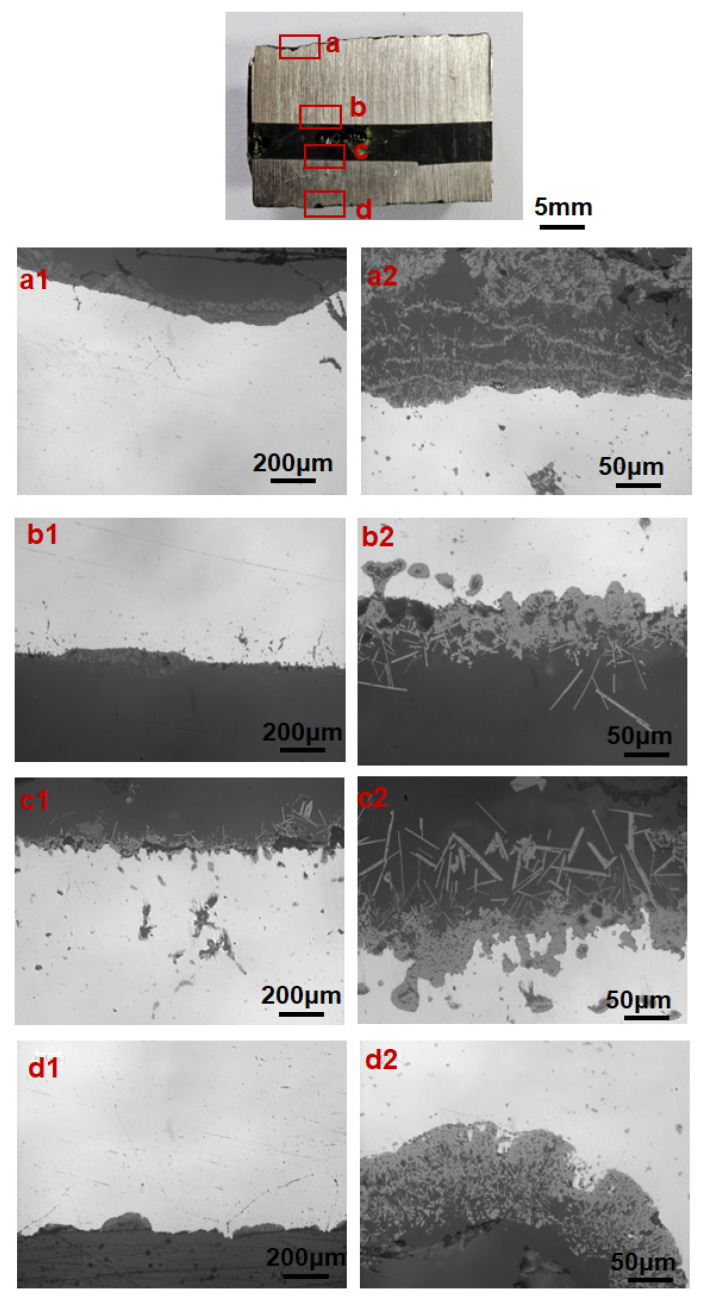
Microstructures for Zone II in bubble tube: (**a**) the outside; (**b**,**c**) the inner wall; (**d**) the inner side. (**a1**,**a2**) microstructures of position (**a**) at different magnifications. (**b1**,**b2**) microstructures of position (**b**) at different magnifications. (**c1**,**c2**) microstructures of position (**c**) at different magnifications. (**d1**,**d2**) microstructures of position (**d**) at different magnifications.

As can be seen from the macroscopic morphology in Figure 5, Zone II as a whole continues the asymmetric corrosion pattern of Zone I (Figure 4). The wall thickness of the upper part (outer side) is relatively well preserved, while that of the lower part (inner side) is significantly thinned, resulting in a different corrosion rate, as shown in Table 2. The inner cavity of the tube is also filled with borosilicate glass (Figure 5(b1–c2)), confirming that melt penetration persists in this area. This macroscopic similarity suggests that the dominant environmental factors leading to asymmetric corrosion, such as flow field distribution and temperature gradient, have a certain spatial continuity from Zone I to Zone II.

**Figure 6:**
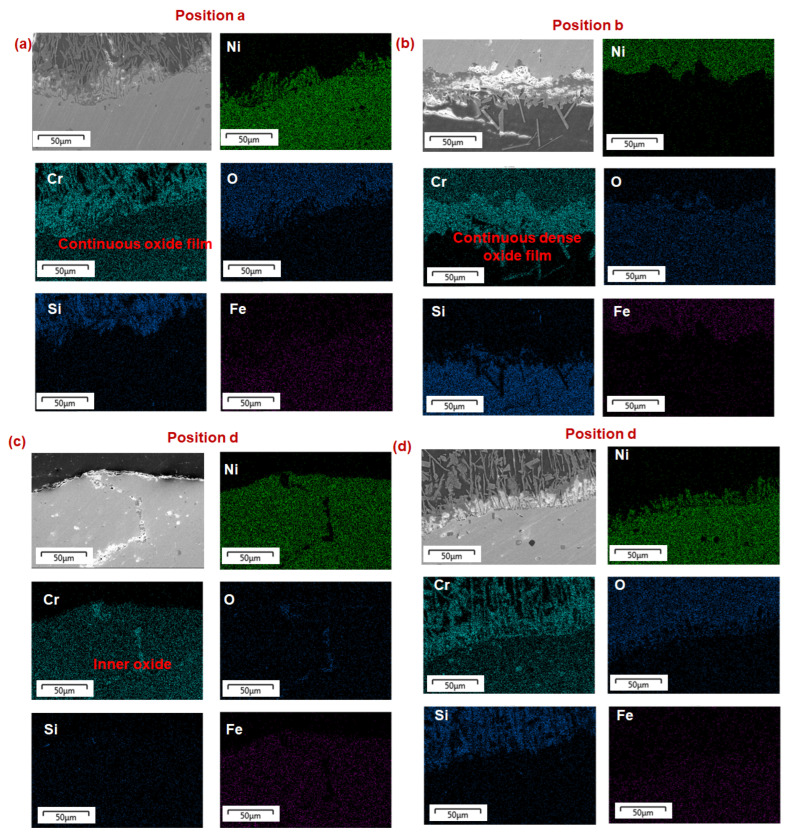
EDS results of the corrosion layers on the bubble tube at Zone II. (**a**) Position a; (**b**) position b; (**c**,**d**) position d.

The EDS results in three positions in Zone II are shown in Figure 6. At the outside of tube in position a (Figure 6a), a continuous oxide film was formed, which offers considerable protection. Meanwhile, some Cr-rich needle-like phases were seen in the borosilicate glass, which means that the Cr-rich oxide film was dissolved into the borosilicate glass. In the inner wall of the tube in position b (Figure 6b), a continuous dense oxide film formed at the interface of the matrix and borosilicate glass. The continuous dense oxide film protected the tube from further corrosion.

For the inner side of the tube in position d (Figure 6c,d), there are two kinds of corrosion behavior. One is the inner oxide without a continuous surface oxide film forming (Figure 6c). Oxidation occurs internally within the matrix, with Cr and O enrichments distributed along paths that likely correspond to grain boundaries or other fast-diffusion channels. This morphology typically arises when the surface oxide film becomes unstable or is mechanically disrupted, allowing oxygen to diffuse inward and form subsurface oxides, leaving the surface unprotected. The other one is the inner oxide uniformly forming amounts of Cr-rich needle-like oxidation products. Meanwhile, the needle-like oxidation products also exist in borosilicate glass.

### 3.4. Corrosion Characteristics for Zone III

Zone III is located at the rear of the bubble tube, near the corner and away from the front air outlet. This area is far from the source of bubble formation and intense disturbance. This zone experiences significantly less intense melt flow, turbulence, and thermal shock than Zone I and Zone II. As a result, the corrosion behavior in Zone III shows different characteristics, reflecting the transition of the material corrosion pattern from “dynamic severe corrosion” to “relatively static high-temperature corrosion” after the environmental harshness has decreased.

**Figure 7:**
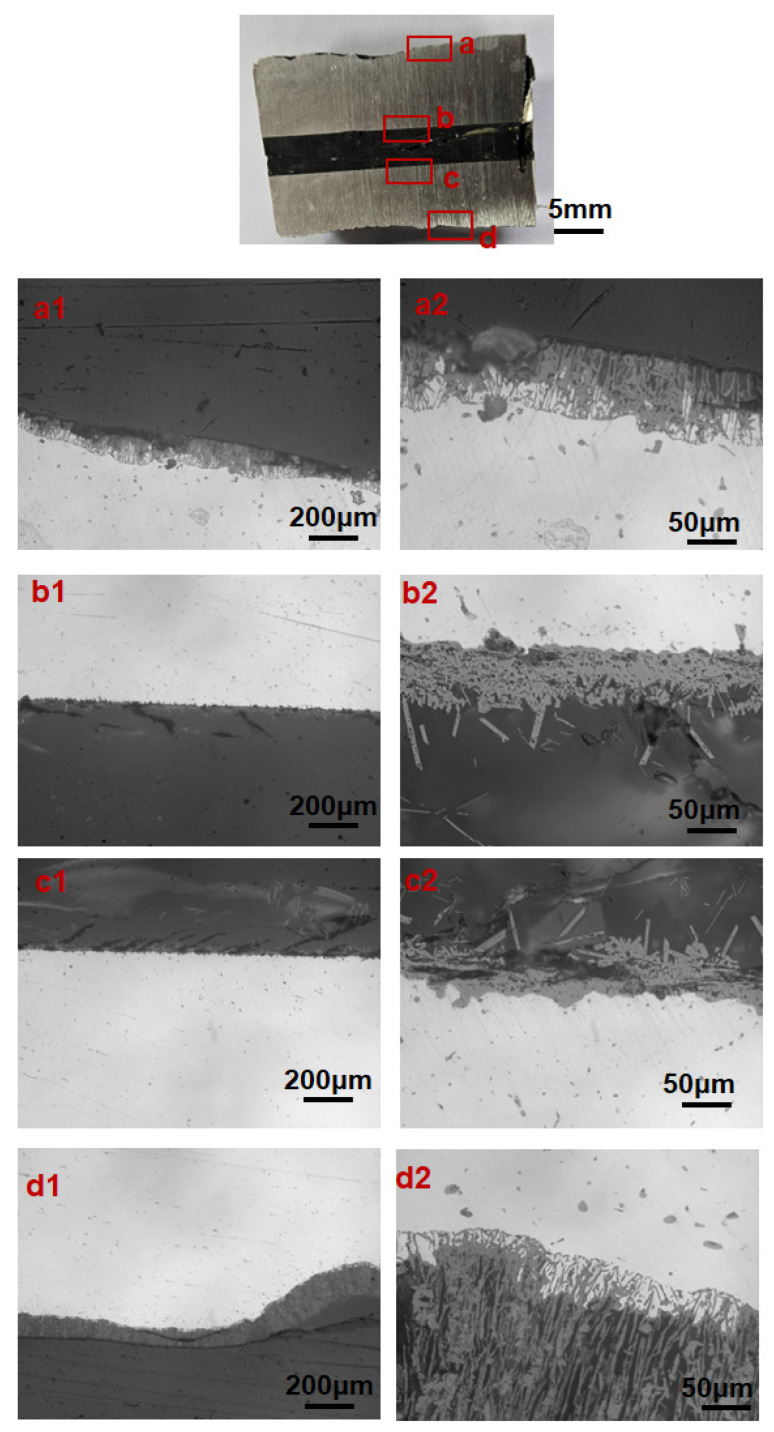
Bubbling tube position III microstructure. (**a**) the outside; (**b**,**c**) the inner wall; (**d**) the inner side. (**a1**,**a2**) microstructures of position (**a**) at different magnifications. (**b1**,**b2**) microstructures of position (**b**) at different magnifications. (**c1**,**c2**) microstructures of position (**c**) at different magnifications. (**d1**,**d2**) microstructures of position (**d**) at different magnifications.

From the macroscopic topography in Figure 7, it can be seen that the asymmetric corrosion of the Zone III section still exists, but the degree has been greatly reduced, only showing that the wall thickness of the upper part (outer side) is slightly thicker than that of the lower part (inner side). The inner cavity of the tube is also completely filled with borosilicate glass, consistent with the front area, confirming the universality of the melt penetration process. In addition, the overall flatness of the outer wall surface is relatively good, which intuitively reflects the weakening of the external fluid’s mechanical scouring effect.

The corrosion features in Zone III (Figure 7), on the outer wall differ notably from those observed in Zones I and II. On the outer curved side (upper half, Figure 7(a1,a2)), the tube wall is covered by a glass layer. The interface undulation is relatively gentle, and plate-like corrosion products are present at the interface, extending further into the glass phase. On the inner curved side (lower half Figure 7(d1,d2)), undulating corrosion pits are visible on the tube wall. These pits are filled with glass, and plate-like products also form at the interface, diffusing into the surrounding glass. In contrast to the frequent discontinuity or destruction of oxide films in Zones I and II, continuous Cr_2_O_3_ oxide films are generally formed on both the upper and lower outer walls of Zone III at the matrix–glass interface. These oxide films effectively block oxygen diffusion into the substrate. They also reduce the oxygen partial pressure within the alloy.

**Figure 8:**
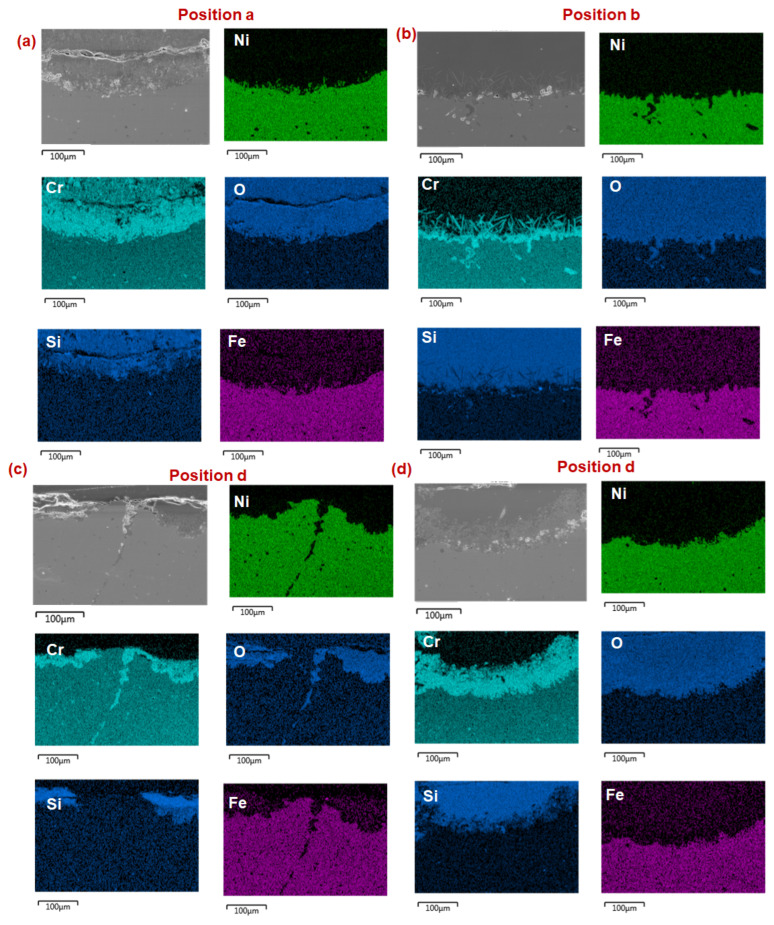
Analysis of corrosion products on the outer wall of the upper tube at bubble tube position III. (**a**) Position a; (**b**) position b; (**c**,**d**) position d.

The EDS elemental distribution maps for Zone III are shown in Figure 8. Distinct corrosion behaviors are observed across the three positions, reflecting the influence of locally varying environmental conditions under the heat–flow coupling effect. On the outside of the tube in position a (Figure 8a), a less continuous oxide film formed, with Cr and O enrichments appearing in localized, patch-like regions. This suggests that oxide film development is incomplete in this area, possibly due to minor local disturbances or microstructural heterogeneity. For the inner wall of the tube in position b (Figure 8b), a continuous and relatively uniform oxide film exhibits as the most protective morphology. The Ni distribution remains intact beneath the film, indicating effective protection. At the inner side of the tube in Zone III, position d (Figure 8c,d) shows similar corrosion behavior as Zones I and II. Differently, an inner oxide formed, as shown in Figure 8c, and a Cr-rich oxide film formed at the interface of the matrix and borosilicate glass. In addition, a thicker oxide layer formed, as shown in Figure 8d, at the outer wall of the tube.

### 3.5. Characteristics of Oxide Layers in Different Zones of Bubble Tube

Quantitative image analysis was performed to measure the thickness of the oxide layers formed on the outer and inner bend sides of the bubble tube in Zones I, II, and III, as summarized in Table 3. In Zone I, the oxide layer on the inner side was too thin and discontinuous to obtain a reliable thickness measurement, whereas on the inner side a measurable layer of approximately 4.94 μm was present. In Zone II, the oxide thickness increased to 1.56 μm on the inner side and 7.53 μm on the outer side. In Zone III, the oxide layers were substantially thicker, reaching 9.28 μm on the inner side and 15.10 μm on the outer side. Across all zones and positions, the chemical composition of the oxide layers was consistently a Cr-rich phase with a Cr:O atomic ratio consistent with Cr_2_O_3_. These results clearly demonstrate that the oxide layer is always thicker on the outer side than on the inner side, reflecting the higher local oxygen partial pressure and stronger melt flow scouring on the bubble-forming side. 

## 4. Discussion

Throughout the microstructure results of Zones I to III from Figure 3, Figure 4, Figure 5, Figure 6, Figure 7 and Figure 8 and Table 2 and Table 3, corrosion on the inner side is consistently more severe than on the outer side, confirming that bubbles rising along the inner bend create higher oxygen partial pressure, flow velocity, and shear stress—direct evidence of the heat–flow coupling effect. The corrosion mechanism during the heat–flow coupled effect is discussed in detail as follows.

### 4.1. Flow-Induced Asymmetry Behavior

**Figure 9:**
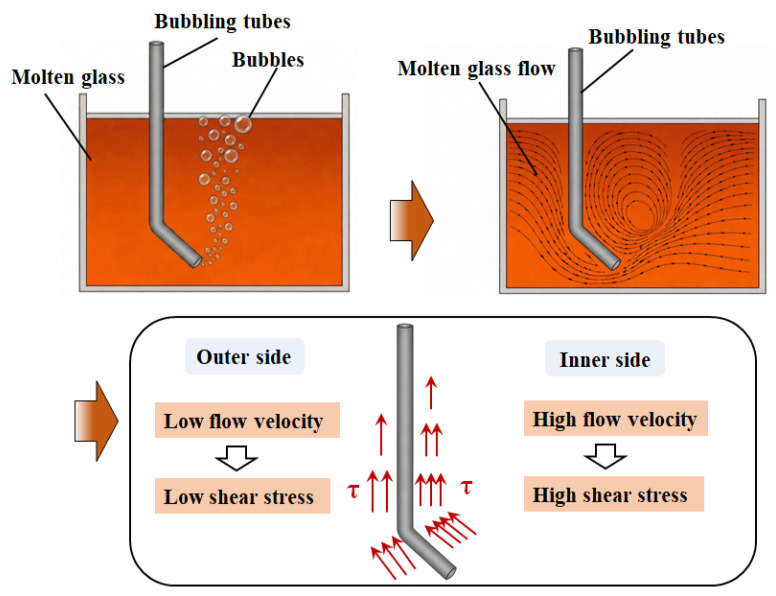
Schematic diagram of the “thermal–flow” field in the service environment of the bubbling tube.

The flow behavior caused by the bubble tube is depicted in a schematic diagram, as shown in Figure 9. Based on the experimental and theoretical framework established by Tihon et al. [28] for bubble rise in inclined channels, the liquid flow dynamics and wall shear stress induced by bubble formation in the furnace can be quantitatively assessed. In the present study, argon gas is introduced through a bubbling tube with an inner diameter of 6 mm at a flow rate of 0.8 L/min at Table 2. Assuming bubble formation frequency of 2–5 bubbles per second, the resulting bubble swarm creates local melt velocities estimated at 0.1–0.5 m/s. According to Tihon et al. [28], the wall shear rate $s_{w}$ in the liquid film separating the bubble from the tube wall can be related to the film thickness $h_{F}$ by the force balance as follows:

(2) $$h_{F}=\frac{\mu }{\rho g\sin\alpha }\cdot s_{w}$$

where μ and ρ are the viscosity and density of the molten glass, respectively, and α is the inclination angle relative to the horizontal.

For the bubble tube in the present configuration, the bubbles preferentially rise along the inner bend side, where the local shear stress is significantly higher. Using the estimated melt velocity *U_F_* ≈ 0.3 m/s in the reverse flow region and assuming a melt viscosity of approximately 10 Pa·s at 1150 °C, the wall shear rate can be approximated from the velocity gradient:

(3) $$s_{w}\approx \frac{U_{F}}{h_{F}}$$

Substituting into the force balance yields an estimated film thickness on the order of 10^−4^ m and wall shear stress $\tau_{w}=\mu s_{w}$ ranging from approximately 30 to 150 Pa, depending on local flow conditions. Furthermore, Tihon et al. [28] demonstrated that for large bubbles in inclined channels, the wall shear stress is unevenly distributed: the maximum reverse flow occurs on the lower wall at intermediate inclinations, while the upper wall experiences significantly lower shear stress. In the present bubbling tube, this asymmetry directly corresponds to the observed corrosion pattern, where the inner bend (lower half) exhibits more severe material loss due to higher oxygen partial pressure and intensified fluid scouring. The scaling of bubble velocity proposed by Tihon et al. [28] further supports the interpretation that the bubble-induced flow is dominated by inertial and gravitational effects, with the bubble terminal velocity approximated by the following equation:

(4) $$U_{B}=0.2\sqrt{gP}$$

where *P* is the channel perimeter.

Although the tube geometry in this study is circular rather than rectangular, the underlying principle—that bubble-induced wall shear stress is governed by flow velocity of molten glass—still holds true. At the outer side of bubble tube, the low flow velocity induced low shear stress. On contract, the high flow velocity induced high shear stress at the inner side of the tube.

### 4.2. Multi-Physical Field Coupling Causes of Corrosion Asymmetry

The common phenomenon of more severe corrosion on the inner curved side was observed in all three regions (Figure 3, Figure 5 and Figure 7, Table 3), which is an inevitable result of the non-uniform distribution of the multi-physics field.

**Figure 10:**
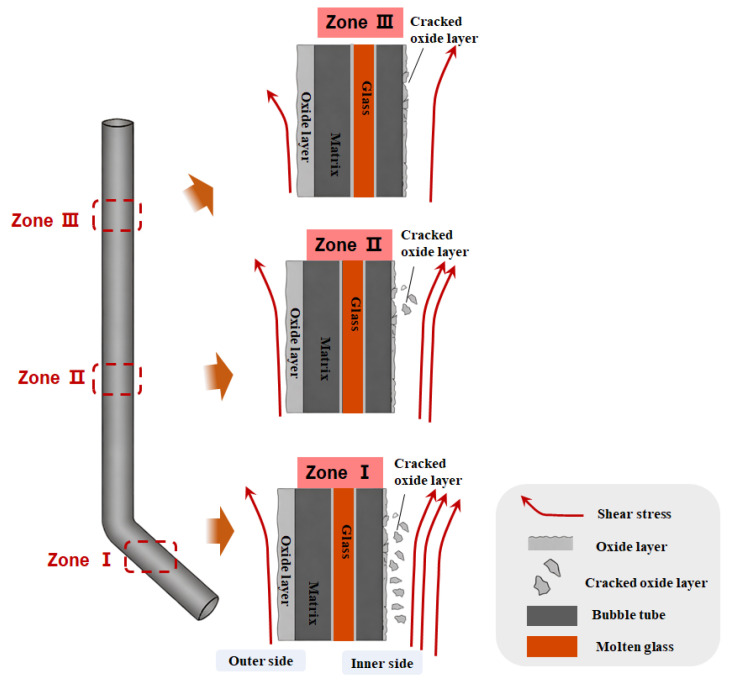
Schematic diagram of the corrosion mechanisms for various zones of the bubbling tube.

The cause can be attributed to three points:

1. Hydrodynamic differences. The melt flow rate is higher and the turbulence is stronger on the inner side (Figure 9), which not only directly intensifies the shear and scouring effect on the oxide film (mechanical factor), but also enhances the mass transfer process of reactants (oxygen) and products (soluble oxides) (chemical factor), accelerating corrosion in a double manner [28,29,30,31,32,33,34,35,36,37].
2. Micro-region differences in temperature field. Intense flow may cause slight temperature inhomogeneity in the circumferential direction of the pipe wall by affecting local heat exchange, and the corrosion rate is extremely sensitive to temperature. The gradual weakening of the asymmetry from Zone I to Zone III, in line with the tendency of the bubble effect to decay with distance, further supports the dominant role of the aforementioned fluid and mass transfer factors [38,39,40,41].
3. The formation and dissolution of oxide films. Oxide film continuity correlates negatively with scouring intensity: Zone I (Figure 4, Table 3) and parts of Zone II (Figure 6, Table 3) exhibit incomplete films prone to spallation, whereas Zone III (Figure 8, Table 3) forms continuous films under milder conditions. Even within Zone III, variations exist—positions b and d show continuous oxide films, while position a displays patchy coverage and position c undergoes internal oxidation. Grain boundary oxidation occurs where films are absent or incomplete, representing an important degradation pathway [19,26,42]. From head (Zone I) to tail (Zone III), the dominant mechanism shifts from erosion-dominated film destruction to high-temperature oxidation with flow as a modifying factor, as shown at Figure 10.

## 5. Conclusions

This study provides the first systematic documentation of corrosion behavior in Joule-heated ceramic melter bubble tubes under actual heat–flow coupling service conditions. Unlike previous studies that investigated high-temperature corrosion or erosion wear in isolation, our results reveal the synergistic intensification mechanism where fluid scouring continuously disrupts oxide film formation, leading to an accelerated oxidation–peeling–reoxidation cycle that cannot be predicted from static corrosion studies alone. The main conclusions were drawn as follows:

(1) Corrosion is asymmetrically distributed along the circumferential direction of the tube. Quantitative wall thickness measurements reveal that the corrosion rate on the inner side (lower half) is approximately two times higher than that on the outer side (upper half), resulting in non-uniform thinning of the tube wall in the most severely affected corrosion regions. This asymmetry is directly attributed to stronger melt scouring on the bubble-forming side.
(2) The Cr-rich oxide film formed on the tube surface provides partial protection to the matrix. However, statistical analysis of oxide layer thickness shows that in areas with intense scouring (inner side of the tube), the film is discontinuous with smaller thickness, whereas in areas with minimal scouring (outside of the tube), a continuous film is maintained with bigger thickness. This quantifies the destructive effect of fluid shear on oxide film stability and demonstrates that protective film integrity is severely compromised under coupled conditions.
(3) A novel finding is the identification of grain boundary oxidation as a critical degradation pathway even in areas without direct glass contact. This internal oxidation network, with Cr-rich oxide precipitates extending along grain boundaries, represents a previously unreported failure mode that may significantly compromise mechanical integrity before macroscopic wall thinning becomes apparent.
(4) Based on these findings, future work should focus on developing alloys with enhanced oxide film stability under dynamic scouring conditions—such as those forming more adherent α-Al_2_O_3_ or multilayer oxide structures—and on optimizing bubbling parameters to reduce local flow velocity and shear forces. Controlled laboratory experiments with quantitative corrosion rate measurements are needed to develop predictive models for bubble tube lifetime under heat–flow coupling conditions.

## Figures and Tables

**Table 1:** Summary of service conditions and operating parameters for bubble tube.

Parameter	Value
Furnace temperature	1150 °C
Glass melt composition	Borosilicate: 55–60% SiO_2_, 15–20% B_2_O_3_, 10–15% Na_2_O, 4–6% Al_2_O_3_, 3–5% CaO, 2–4% others
Immersion depth	~800 mm
Tube initial wall thickness	~12 mm
Tube material composition	28–30% Cr, 9–11% Fe, 0.3% Si, 0.2% Mn, 0.02% C and balance Ni (in wt.%)
Argon flow rate	0.6~1.0 L/min
Argon inlet pressure	0.15~0.30 MPa
Service duration	30 days (720 h)

**Table 2:** Average corrosion rates in the different positions of bubble tube.

Position	Corrosion Rate (mm/Day)	
	Outer Side	Inner Side
Zone I	0.047	0.118
Zone II	0.079	0.154
Zone III	0.028	0.083

**Table 3:** The thickness and of oxide layers in the different positions of bubble tube.

Position	Thickness of the Oxide Layers (μm)		Chemical Compositions
	Inner Side	Outer Side	
Zone I	/	4.94	Cr_2_O_3_
Zone II	1.56	7.53	Cr_2_O_3_
Zone III	9.28	15.10	Cr_2_O_3_

## Data Availability

The original contributions presented in this study are included in the article. Further inquiries can be directed to the corresponding authors.
